# Ginger Stimulates Hematopoiesis via Bmp Pathway in Zebrafish

**DOI:** 10.1371/journal.pone.0039327

**Published:** 2012-06-25

**Authors:** Karine F. Ferri-Lagneau, Karni S. Moshal, Matthew Grimes, Braden Zahora, Lishuang Lv, Shengmin Sang, TinChung Leung

**Affiliations:** 1 The Biomedical/Biotechnology Research Institute, North Carolina Central University, North Carolina Research Campus, Kannapolis, North Carolina, United States of America; 2 Center for Excellence in Post-Harvest Technologies, North Carolina Agricultural and Technical State University, North Carolina Research Campus, Kannapolis, North Carolina, United States of America; 3 Department of Biology, North Carolina Central University, Durham, North Carolina, United States of America; University of Sheffield, United Kingdom

## Abstract

**Background:**

Anemia is a hematologic disorder with decreased number of erythrocytes. Erythropoiesis, the process by which red blood cells differentiate, are conserved in humans, mice and zebrafish. The only known agents available to treat pathological anemia are erythropoietin and its biologic derivatives. However, erythropoietin therapy elicits unwanted side-effects, high cost and intravenous or subcutaneous injection, warranting the development of a more cost effective and non-peptide alternative. Ginger (*Zingiber officinale*) has been widely used in traditional medicine; however, to date there is no scientific research documenting the potential of ginger to stimulate hematopoiesis.

**Methodology/Principal Findings:**

Here, we utilized *gata1:dsRed* transgenic zebrafish embryos to investigate the effect of ginger extract on hematopoiesis in vivo and we identified its bioactive component, 10-gingerol. We confirmed that ginger and 10-gingerol promote the expression of *gata1* in erythroid cells and increase the expression of hematopoietic progenitor markers *cmyb* and *scl*. We also demonstrated that ginger and 10-gingerol can promote the hematopoietic recovery from acute hemolytic anemia in zebrafish, by quantifying the number of circulating erythroid cells in the dorsal aorta using video microscopy. We found that ginger and 10-gingerol treatment during gastrulation results in an increase of *bmp2b* and *bmp7a* expression, and their downstream effectors, *gata2* and *eve1*. At later stages ginger and 10-gingerol can induce *bmp2b*/*7a*, *cmyb*, *scl* and *lmo2* expression in the caudal hematopoietic tissue area. We further confirmed that Bmp/Smad pathway mediates this hematopoiesis promoting effect of ginger by using the Bmp-activated Bmp type I receptor kinase inhibitors dorsomorphin, LND193189 and DMH1.

**Conclusions/Significance:**

Our study provides a strong foundation to further evaluate the molecular mechanism of ginger and its bioactive components during hematopoiesis and to investigate their effects in adults. Our results will provide the basis for future research into the effect of ginger during mammalian hematopoiesis to develop novel erythropoiesis promoting agents.

## Introduction

The bone morphogenetic protein (Bmp) signaling pathway plays a critical role in hematopoeisis during the induction and maintenance of Hematopoietic Stem Cells (HSCs) in the “Aorta-Gonad-Mesonephros” (AGM) axis [1]–[2]. Bmp’s are members of the TGF-β superfamily of secreted factors, which regulate the development of multiple organ systems, such as bone, neural and renal tissue. In addition to their function in dorsal-ventral specification, Bmp’s regulate the development of human HSCs [3] and embryonic hematopoiesis (blood cell formation) during early vertebrate development, but this function is independent of their mesoderm inductive activity [4]. In zebrafish, *bmp2b*, *bmp4* and *bmp7a* expression is especially important for ventral mesoderm patterning [5]–[7] and blood specification [8]–[9]. Bmp signaling is required to initiate the HSC program at the floor of the dorsal aorta and to maintain normal levels of HSC descendants during hematopoeisis [10]–[11]. In mammals, the blood cells originate in the blood islands of the yolk before they are produced in the body of the embryo [12]. In adults, the bone marrow is the primary tissue for hematopoeisis and erythropoiesis, with blood cells originating from stem cells; however, the molecular nature of this process is not well understood [13]. Similarly, in the vertebrate zebrafish, blood cells form in different sites during early embryonic development starting from the mesoderm near the aorta (ICM or Intermediate Cell Mass) and then at the posterior blood island (PBI) in the tail. These sites are of special interest because they contain hematopoietic progenitors which give rise to the blood cells and can be used as a model to study the molecular mechanism of hematopoeisis and erythropoiesis in vivo [12]–[13].

The AGM, arising from the mesodermal primary cell layer, is the main site for hematopoeisis in mammals [14], and the addition of Bmp to long term cultures of AGM-derived HSCs increases their growth and survival [15]. The zebrafish equivalents of these tissues, arising also from the mesoderm, are the ICM and the PBI, where the hematopoietic progenitor markers *cmyb*, *scl* and *lmo2* are expressed during development [16]–[22]. In both mammals and zebrafish, hematopoeisis occurs in two distinct steps, the ‘primitive’ and ‘definitive’ waves. The ICM and PBI represent the site of ‘primitive’ or first wave of hematopoeisis. The ICM contains hemangioblasts, which can differentiate into pro-erythroblasts or angioblasts (blood/vessel precursors), whereas the PBI generates erythro-myeloid precursors, including pro-erythroblasts and myeloblasts [16]. The zinc finger transcription factor *GATA-binding protein 1* (*gata1*) is a master regulator of erythrocyte commitment and maintenance [23]. *gata1* and *spleen focus forming virus proviral integration oncogene* (*spi1/pu1*) determine the erythroid vs. myeloid cell fates respectively to maintain balance of both cell lineages [24]. In mice, the subsequent ‘definitive’ wave of hematopoiesis gives rise to hematopoietic stem cells (HSCs) capable of differentiating into any of the blood cell lineages; hematopoiesis takes place in both the aorta-gonad-mesonephros (AGM) region and the umbilical vessels at embryonic stage E10–11 [14], [25]. As development progresses, erythropoiesis gradually shifts from the spleen and liver of the fetus to the bone marrow in mammals [13], equivalent to the kidney marrow in zebrafish. Real-time observations in live reporter transgenic animals have confirmed that the transition from hemogenic endothelium in the ventral wall of the aorta to HSCs actually occurs in the mouse, zebrafish and *Xenopus* [18]–[19], [25]–[26]. In zebrafish, the definitive wave of hematopoiesis occurs in the kidney marrow and thymus after a transient development in the PBI-derived caudal hematopoietic tissue (CHT) and the hemogenic endothelium in the ventral wall of the dorsal aorta [17], [27]. Stage-specific transcription during definitive hematopoiesis is driven by *runt-related transcription factor 1* (*runx1*) and *avian myeloblastosis viral oncogene homolog* (*cmyb*) [28].

The erythropoiesis-stimulating agents available to treat pathological anemia, commonly associated with end stage renal disease and cancer chemotherapy, such as Aranesp, Procrit, Epogen and Neorecormon, are biologic derivatives or various formulations derived from the same protein, erythropoietin (EPO). However, the side effect of using EPO therapy includes life-threatening cardiovascular complications. Another drawback of using EPO and its analogs is the high cost and the injectable mode of delivery, therefore warranting the development of a non-peptide alternative. Here, we identified a natural product, namely ginger (*Zingiber officinale*), which can stimulate hematopoiesis in zebrafish embryos. By using a chemically inducible hemolytic anemia model, we showed that ginger extract and its active component 10-gingerol (10-G) can promote hematopoietic recovery in a process that is mediated via the *bmp* signaling pathway.

## Results

### Ginger (*Zingiber officinale*) and its Bioactive Components

Ginger is widely used as both a spice and an herbal medicine for rheumatism, nausea, colds and flu, diarrhea, muscular disorders, dyspepsia, poor appetite and diabetes [29]. Gingerols, the major phenolic components of ginger [30] and shogaols, the dehydrated forms of gingerols, possess anti-inflammatory and anti-cancer properties [29], [31]–[32]. In the present study, we purified various gingerols and shogaols using previously published methods with slight modifications [31] and confirmed their structures by ^1^H and ^13^C NMR analysis (Figure S1). Ginger extract and the purified 6-, 8-, 10-gingerol (6-G, 8-G, 10-G) and 6-, 8-, 10-shogaol (6-S, 8-S, 10-S) were used in the following experiments to evaluate their potential to promote hematopoiesis in the zebrafish model.

### Ginger Promotes *Gata1* Expression

**Figure 1:**
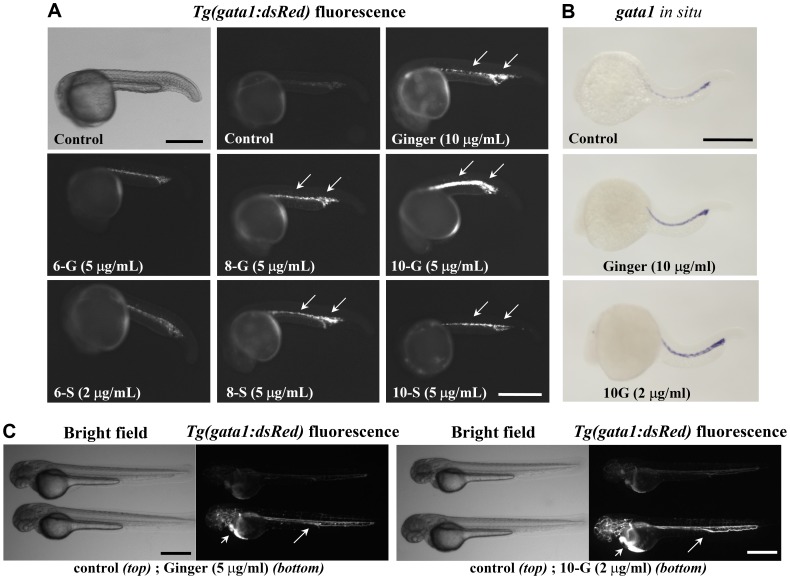
Ginger extract and its purified phenolic compounds promote *Tg(gata1:dsRed)* fluorescence and *gata1* mRNA expression. (A) Bright field (top left) and *Tg(gata1:dsRed)* fluorescence of zebrafish embryos at about 22 hpf, before the onset of circulation (anterior to the left). Exposure to ginger extract or its compounds 8-gingerol (8-G), 10-gingerol (10-G), 8-shogaol (8-S) and 10-shogaol (10-S) promoted *Tg(gata1:dsRed)* fluorescent erythroid cell development in the ICM and PBI (arrows), as compared to control embryos. N = 35 embryos per group. In this panel, we show an embryo treated with a lower concentration of 6-S (2 µg/ml) as this compound was toxic at higher doses. Scale bar = 400 µm. (B) Whole-mount in situ hybridization of ginger or 10-G treated embryos (8 hpf to 21 hpf exposure) revealed increased expression of *gata1* transcript at 22 hpf. N = 50 embryos per group. Scale bar = 350 µm. (C) At 48 hpf, control embryos at the top; ginger or 10-G treated embryos at the bottom. Scale bar = 500 µm. Fluorescent erythrocytes circulating in the axial vasculature (arrows) and in the pericardial space (arrow heads).

**Figure 2:**
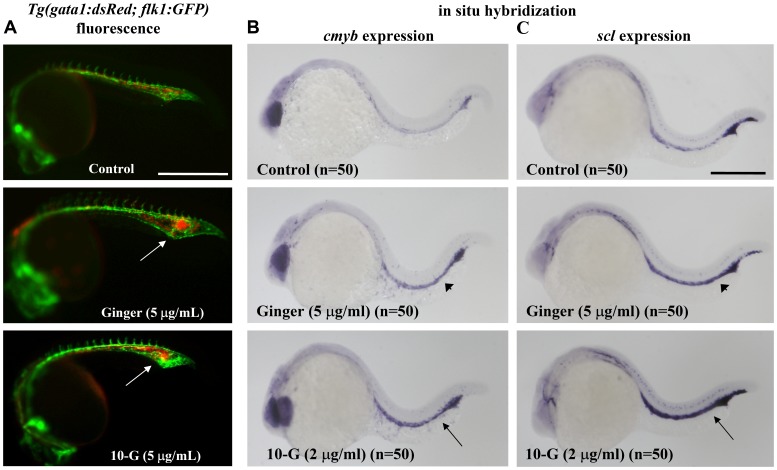
Ginger/10-G treatment increases hematopoietic progenitor markers expression. Zebrafish embryos were treated with ginger or 10-G from 9 to 21 hpf. (A) *Tg(gata1:dsRed)* for erythrocyte and *Tg(flk1:GFP)* for blood vessels, double-fluorescent overlay pictures of embryos at 22 hpf after exposure to ginger or 10-G. Hypertrophy of the PBI vascular plexus in *Tg(flk1:GFP)* after ginger or 10-G treatment, with *Tg(gata1:dsRed)* red fluorescent erythrocytes accumulated inside the honeycomb-like vasculature (arrows). Scale bars = 500 µm. Whole-mount in situ hybridization of *c-myb* (B) and *scl* (C) in zebrafish embryos at 22 hpf. Both hematopoietic progenitor markers were up-regulated in primitive hematopoietic tissues (ICM+PBI) upon ginger (arrow head) or 10-G (arrow) exposure. Scale bar = 350 µm.

The GATA-binding factor 1 (Gata1), a zinc finger transcription factor, is an early marker and key regulator of erythropoiesis. Erythrocytes can be visualized *in vivo* in *Tg(gata1:dsRed)* transgenic zebrafish embryos by fluorescence microscopy as they exhibit an erythrocyte-specific red fluorescence under the control of the *gata1* promoter [33]. Here, we studied the hematopoeisis promoting effect of ginger extract and its components 6-, 8-, and 10-gingerol and 6-, 8-, and 10-shogaol in zebrafish embryos from the late gastrulation stage at 9 hour-post-fertilization (hpf) to the 21 hpf stage before the onset of circulation. Figure 1 illustrates that treatment with ginger extract or its components, including 8-G, 10-G, 8-S and 10-S, resulted in increased fluorescence intensity of *Tg(gata1:dsRed)* transgenic expression at 1 day-post-fertilization (dpf) (Figure 1A), both in the ICM and the PBI. In addition, whole-mount in situ hybridization analyses using a specific *gata1* anti-sense RNA probe confirmed that exposure of *Tg(gata1:dsRed)* zebrafish embryos to ginger extract (5–10 µg/ml) and 10-G (as low as 2 µg/ml) promoted *gata1* expression in developing erythrocytes (Figure 1B). At 2 dpf, when the heart has begun to beat rhythmically and blood circulation is established, we observed an increase in *gata1:dsRed* fluorescence signal in circulating erythroid cells within the vasculature, especially in zebrafish treated with 5 µg/ml ginger and 2 µg/ml 10-G (Figure 1C). These results suggest that ginger extract (5–10 µg/ml) and its components 8-G, 10-G, 8-S and 10-S (2–5 µg/ml) potentially stimulate hematopoiesis. Our data identified 10-G as the most potent bioactive component of ginger extract in promoting the primitive wave of erythropoiesis and the least toxic to early developing zebrafish embryos. In Figure 2A, pictures of double transgenic *Tg(flk1:GFP);Tg(gata1:dsRed)* embryos show the expansion of the PBI caudal region, where a cavity has emerged surrounded by a hypertrophic vascular plexus and filled with *Tg(gata1:dsRed)* erythrocyte progenitor cells at 22 hpf, following exposure to ginger extract (5 µg/ml) or 10-G (5 µg/ml). After the establishment of circulation, the morphology of the PBI of these treated embryos is indistinguishable from their untreated siblings, although they have much more circulating erythrocytes (Figure 1C). These data provide evidence that ginger and its bioactive components could potentially stimulate erythropoiesis during the primitive wave of hematopoiesis in early developing zebrafish embryos.

To further delineate the effect of ginger in promoting erythrocyte differentiation, we analyzed the effect of ginger on a mouse erythroblast cell line (ATCC-TIB55/BB88) *in vitro*. Figure S2 shows that the mouse erythroblasts remained undifferentiated in the control conditions of 0–0.05% DMSO. On the other hand, ginger treatment (5–20 µg/ml) significantly promoted erythrocyte differentiation of mouse erythroblasts as we detected the production of hemoglobin using benzidine staining [34]. A similar level of cell viability was obtained in all treatments after 5 days (70–80% viable cells; unpublished data), using trypan blue staining. High concentration of ginger (20 µg/ml) for 5 days induced differentiation of erythrocytes (3.3%) in contrast to 0% in control. At the same time, the treatment with ginger led to a reduction in the number of proliferating cells as compared to the control (24.3%; Figure S2). The effect of ginger is dose dependent, as we observed fewer (0.93%) differentiated erythrocytes, and a significant increase in the number of proliferating cells (13.3%; Figure S2) at lower concentration of ginger (5 µg/ml). Overall, the effect of ginger on erythrocyte differentiation *in vitro* is not significant enough to account for the increase in the number of erythroid cells induced by ginger *in vivo*. For this reason, we further investigated the effect of ginger on hematopoietic progenitor cells *in vivo*.

### Ginger Promotes Expression of Hematopoietic Progenitor Markers

Like all vertebrate organisms, zebrafish show waves of hematopoiesis during development [12]. Zebrafish hematopoiesis originates from the *cmyb*-positive primitive hematopoietic progenitors arising in the ventral mesoderm-derived ICM/PBI [23]. In 48 hpf embryos, cells expressing *cmyb* are scattered among the first progenitors of definitive hematopoiesis along the ventral wall of the dorsal aorta; in zebrafish, this thin mesenchyme between the dorsal aorta and the posterior cardinal vein corresponds to the mammalian AGM [5]–[6], [27]. At 4 dpf, *cmyb* is weakly expressed in the trunk and tail in hematopoietic clusters, but by 5 dpf, it is mainly expressed in the caudal vein plexus (CHT), pronephric glomeruli and thymi.

The CHT reaches its maximal activity by 5–6 dpf, although it remains hematopoietic until at least 14 dpf, associated with the definitive wave in the caudal vein plexus and supporting proliferation and differentiation of blood precursors. To analyze the effect of ginger on hematopoietic progenitors, we used whole-mount in situ hybridization to detect the expression of *cmyb* transcription factor (a marker of immature hematopoietic cells whose expression decreases as these cells differentiate) and *stem cell leukemia hematopoietic transcription factor*, also named *T-cell acute lymphocytic leukemia 1 (scl/tal1,* a marker for hemangioblasts, already fated to become hematopoietic cells). Figure 2 shows that exposing late gastrulation (9 hpf) embryos to ginger (5 µg/ml) or its bioactive component 10-G (2 µg/ml) up-regulates the expression levels of hematopoietic progenitor markers, such as *cmyb* (Figure 2B) and *scl/tal1* (Figure 2C), in the ICM/PBI region at 22 hpf. These data suggest that ginger or 10-G treatments could increase primitive erythropoiesis in zebrafish embryos through the promotion of the hematopoietic progenitor cell numbers.

### Mechanistic Insight: Ginger Induces *bmp7a/2b* Expression

**Figure 3:**
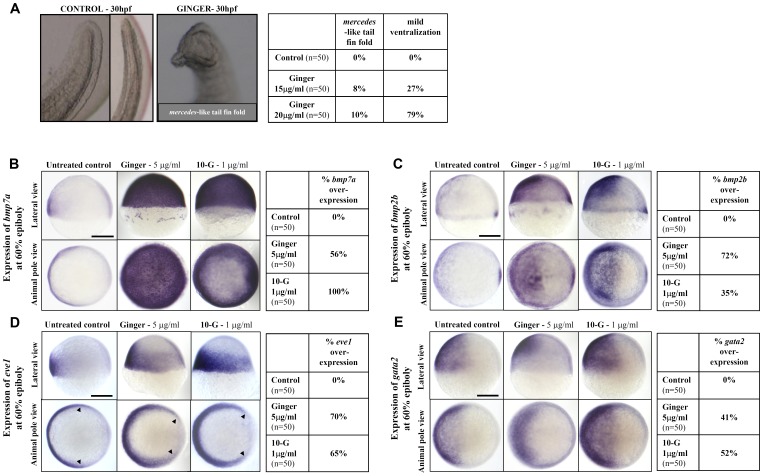
Ginger/10-G treatment during gastrulation promotes *bmp2b/7a* and Bmp target gene expression in zebrafish embryos. (A) Treatment of late gastrulae with ginger at 15 or 20 µg/ml induces the *mercedes* mutant-like phenotype (partial duplication of the tail fin) at 1 dpf in 8% or 10% of the treated embryos, respectively. Thus, the zebrafish embryos exposed to ginger extract mimic the phenotype of the *ogon* mutant, which has a mutation in *sizzled*, a *bmp* suppressor gene, at 1 dpf. (B) *bmp7a* expression was strongly increased and extended to the entire blastoderm at 60% epiboly, following short-term exposure to ginger (5 µg/ml) or 10-G (1 µg/ml) from sphere (4 hpf) to 60% epiboly (7 hpf) stages. (C) Up-regulation and extension of the expression domain were observed for *bmp2b* at 60% epiboly. (D–E) Accordingly, BMP target genes were up-regulated after ginger/10G treatment from the sphere stage (4 hpf) to 7 hpf, as illustrated by enhanced *eve1* extended towards the dorsal side (arrow heads), a ventral mesoderm marker (D), and *gata2,* a non-neural ectoderm marker (E), in zebrafish embryos at 60% epiboly. Pictures on left panels show gastrulae, dorsal side to the right (B–E) and statistics tables (right panels) are representative of three independent experiments. N = number of embryos per group. Scale bars = 250 µm.

When zebrafish embryos were treated during early development with high concentrations of ginger, i.e. 15 to 20 µg/ml from the shield stage (6 hpf) to 1 dpf, we observed a severe defect with *mercedes* mutant-like tail phenotype, characterized by a partial duplication of the tail fin [35]–[36] (Figure 3A) and ventralization of embryos, exhibiting a swollen yolk sac extension with excessive *Tg(gata1:dsRed)* fluorescent erythrocytes accumulating in the ICM/PBI before the onset of blood circulation. Most importantly, these observed phenotypes are reminiscent of an enhanced Bmp activity during early development.

To determine whether ginger modulates *bmp/smad* signaling, we used whole-mount in situ hybridization to analyze temporal-spatial gene expression of *bmp2b*, *bmp4* and *bmp7a*, following ginger or 10-G treatment of zebrafish embryos. As shown in Figure 3B, early short-term (3-hour) exposure of zebrafish embryos during gastrulation to ginger extract (5 µg/ml) and 10-G (1 µg/ml) from the sphere stage (4 hpf) to 60% epiboly stage (7 hpf), increased the level and extended the domain of *bmp7a* expression. At this stage *bmp7a* expression is normally restricted to the ventral side of the blastoderm, but ginger and 10-G treatments induced expression throughout the entire blastoderm. In addition, ginger exposure during gastrulation (but not 10-G) results in a small delay in the progression of embryonic cell epiboly, whereas the epibolic movement of the yolk syncytial layer is not affected (Figure 3B–D).

As shown in Figure 3C, early treatment of embryos (from the sphere stage) with ginger or 10-G also resulted in an increase in the expression of *bmp2b* and expanded its expression domain towards the dorsal side at 60% epiboly stage, but did not induce the intense global expression seen with *bmp7a*. We observed no change in *bmp4* expression in response to ginger or 10-G exposure (Figure S3).

To further delineate the *bmp* signal axis, we determined the expression of *bmp* target genes *even-skipped-like1* (*eve1,* a ventral mesodermal marker) [35] and *GATA-binding protein 2* (*gata2,* a non-neural ectodermal marker) [37] and observed an increase in their mRNA levels, following early exposure to ginger (5 µg/ml) or 10-G (1 µg/ml) from sphere (4 hpf) to 60% epiboly (7 hpf) stages (Figure 3D–E). Hence, we provide evidence that treatment of early embryos with ginger extract or 10-G, one of its individual active components, increases the expression of *bmp2b/7a* and two *bmp* target genes, *eve1 and gata2*.

The *bmp* signaling pathway is regulated by the action of dorsalizing signals from extracellular protein factors coded by genes such as *chordin (chd)* [38], and *fibroblast growth factor8* (*fgf8*) and *bmp* signaling pathways interact during ventral mesoderm patterning in blood formation [39]; therefore, we analyzed the expression of *chd* and *fgf8* in early stage embryos. No change in their transcript levels was seen following exposure to ginger extract from 3 hpf to shield (6 hpf) stages (Figure S4). Our results are consistent with a previous study by Miller-Bertoglio and colleagues who had shown that *chd* expression is indistinguishable in weakly ventralized *mercedes* mutant embryos at 75% epiboly and their wildtype siblings [38]. Thus, the hematopoietic effect of ginger is independent of the mRNA expression of *chd* at the dorsal organizer and *fgf8* at the dorsal and ventral margins from 3 hpf to shield stages (Figure S4).

### Ginger Induces *bmp7a/2b* Expression in the CHT Region

**Figure 4:**
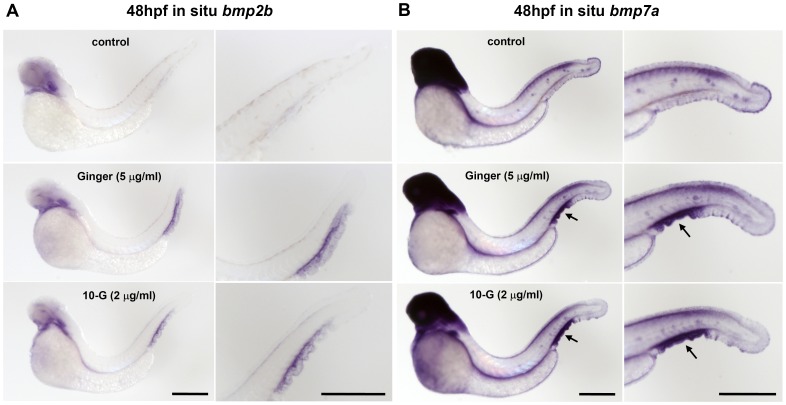
Ginger/10-G treatment after gastrulation promotes *bmp2b/7a* in the developing caudal hematopoietic tissue. (A–B) Zebrafish embryos were treated with ginger (5 µg/ml) or 10-G (2 µg/ml) from 10 to 48 hpf, followed by whole-mount in situ hybridization of *bmp2b* (A) and *bmp7a* (B). Both *bmp2b* and *bmp7a* were up-regulated locally in the CHT (and underlying fin) upon ginger or 10-G exposure (whereas they are not expressed in the CHT of control embryos at 48 hpf). Scale bars = 700 µm.

In order to separate the hematopoietic and mesoderm inductive effects of ginger/10-G on the *bmp* signaling during early embryonic development, we investigated the *bmp2b/7a* expression profiles following exposure to ginger/10-G, from 10 hpf to 48 hpf (Figure 4). In untreated control embryos, *bmp2b* is weakly expressed in the ventral fin epidermis, the ventral posterior mesoderm (hematopoietic), the caudal vein and the posterior cardinal vein during the primitive wave of hematopoiesis. The exposure of embryos to ginger/10-G, starting after the completion of gastrulation, triggers an up-regulation of *bmp2b* expression only in these ventral posterior tissues of the future CHT at 32 hpf (not shown). The contrast in *bmp2b* expression patterns between control embryos and ginger/10-G-treated embryos is even more outstanding at 48 hpf, when the *bmp2b* expression in the ventral posterior mesoderm has become down-regulated in the untreated control embryos, which show no expression in the CHT area, unlike the ginger/10-G treated embryos which exhibit an over expression of *bmp2b* restricted to the CHT region and the corresponding portion of the caudal ventral fin (Figure 4A). We also analyzed the expression patterns of *bmp7a* at 48 hpf after ginger or 10-G exposure and observed its up-regulation in the same region including the CHT and the underlying fin (Figure 4B). Thus, during the transition from the primitive to the definitive wave of hematopoiesis, exposure of zebrafish embryos to ginger extract (5 µg/ml) or 10-G (2 µg/ml) locally up-regulates the expression of *bmp2b* and *bmp7a* in the area of the developing hematopoietic tissue.

### Bmp Inhibitors Block Ginger-induced *bmp* Expression

We next screened bioactive small molecules known to inhibit Bmp/Smad signaling, such as dorsomorphin, LDN193189 and DMH1, to investigate if the ginger-mediated increase in *bmp2b/7a* transcription in the CHT region can be repressed by these specific signaling inhibitors through the Bmp auto-regulatory loop. Figure S5-A shows *bmp2b* expression at 48 hpf after treatment from 10 hpf with ginger alone *versus* combinations of ginger and inhibitors of Bmp/smad signaling, following ranges of concentrations in accordance with their documented specificities in the literature. For instance, Yu and colleagues used 10 µM dorsomorphin to induce a spectrum of dorsalization phenotypes in zebrafish embryos, which vary with the developmental stage of treatment, by specifically antagonizing receptors ALK2/3/6 and not AMPK or VEGFR2 signaling. They also show that dorsomorphin preferentially inhibits BMP/Smad over MAPK p38, TGF-β and Activin signaling [40]. We tested a range of concentrations for combining dorsomorphin (0.1–10 µM) with ginger, and determined that 0.1 µM was sufficient to inhibit the local over-expression of *bmp2b* in the CHT area mediated by ginger (Figure S5). We also tested the dorsomorphin analogues LDN-193189 and DMH-1 using a narrower range of concentrations (0.1–1 µM), as both molecules were shown to be more selective and more potent in blocking Bmp/Smad signaling, without interfering with VEGF or TGF-β signaling [41]–[42]. As shown in Figure S5, 0.1 µM DMH-1 highly antagonized the ginger-induced *bmp2b* over-expression in the CHT region. In this assay, the Bmp type I receptor antagonists DMH-1 and dorsomorphin were slightly more potent than the LDN-193189 analogue in blocking the effect of ginger (Figure S5, bottom table). We observed similar results by analyzing *bmp7a* expression profiles in 48 hpf embryos in the same Bmp type I receptor antagonist screening assay (Figure S6). Altogether, these results suggest that ginger and 10-G can induce *bmp* expression specifically in the ventral tail area including the developing caudal hematopoietic tissue.

### Ginger Enhances Hematopoietic Recovery from Anemia

**Figure 5:**
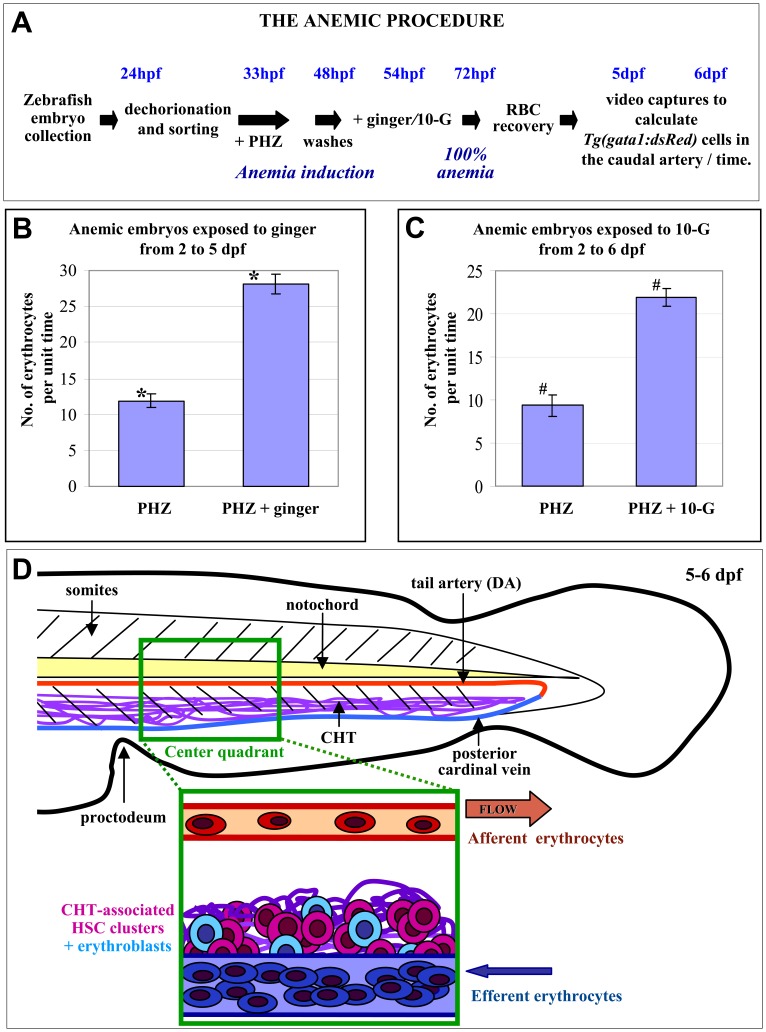
Ginger/10-G treatment increases circulating erythrocytes in anemic zebrafish. (A) A schematic representing the timing and experimental protocol of the anemic procedure, which is required for accurate measurements of the number of circulating erythroid cells within the caudal dorsal aorta. (B–C) Quantitation of *Tg(gata1:dsRed)* fluorescent erythroid cells, within the caudal artery, in anemic zebrafish embryos with ginger treatment from 2 to 5 dpf (B) or 10-G treatment from 2 to 6 dpf (C), respectively (See Figure 6 A and B for detailed quantitative analyses). (D) A cartoon illustrating the tail region filmed for quantitative analyses is shown**.** Note the morphological difference between erythroblasts (round progenitors, light blue-colored) and mature erythrocytes (elongated cells, dark blue-colored). The green box represents the filmed area for quantitative measurements of circulating erythrocytes, with dimensions: 327 µm x 246 µm. Data are represented as mean ± SEM. *p* values were determined by using the Student′s t-test. *, ^#^ represent statistically significant values of *p* = 1.5×10^−10^ and 9.4×10^−6^, respectively.

To further demonstrate that ginger and 10-G stimulate hematopoiesis in a quantitative manner, we established a protocol to measure the number of erythrocytes circulating in the caudal artery (Videos S1 and S2) during hematopoietic recovery (Figure 5A and D) using a phenylhydrazine-inducible hemolytic anemia zebrafish model [6]. Transgenic *Tg(gata1:dsRed)* zebrafish embryos were exposed to 0.5 µg/ml phenylhydrazine from 33 hpf to 48 hpf and then washed extensively (Videos S3 and S5). The anemic embryos were subsequently treated with ginger extract (Video S4) or 10-G (Video S6) starting at 54 hpf. Videos of erythrocytes circulating within the caudal artery, in a portion of the dorsal aorta in the tail region beyond the proctodeum, were acquired at 5 and 6 dpf using a fluorescent microscope (see Methods for detail). For each video, we counted the number of *Tg(gata1:dsRed)* cells entering and exiting the filmed portion of the dorsal aorta. The average was calculated and subsequently corrected by the blood flow ratio. Video analyses indicated that exposure to ginger extract (2 µg/ml) (Figure 5B) produced a 2.4-fold increase in the number of circulating *Tg(gata1:dsRed)* cells compared to phenylhydrazine controls at 5 dpf. When the embryos were treated with the ginger component 10-G (1 µg/ml), we observed a 2.3-fold increase in circulating blood cells (Figure 5C) compared to phenylhydrazine controls at 6 dpf. Therefore, exposure to ginger or 10-G promotes recovery from phenylhydrazine-induced acute hemolytic anemia in zebrafish by increasing the number of circulating *Tg(gata1:dsRed)* erythroid cells. As compared to normal control zebrafish embryos (Video S7), phenylhydrazine treatment completely eliminated circulating *Tg(gata1:dsRed)* cells at 3 dpf (Video S8); therefore, erythrocytes produced/recovered from phenylhydrazine treatment after 3 dpf were most likely derived from erythroid progenitors (erythroblasts) generated by definitive hematopoietic tissues.

### Bmp Signaling is Essential for Ginger-induced Hematopoietic Recovery

**Figure 6:**
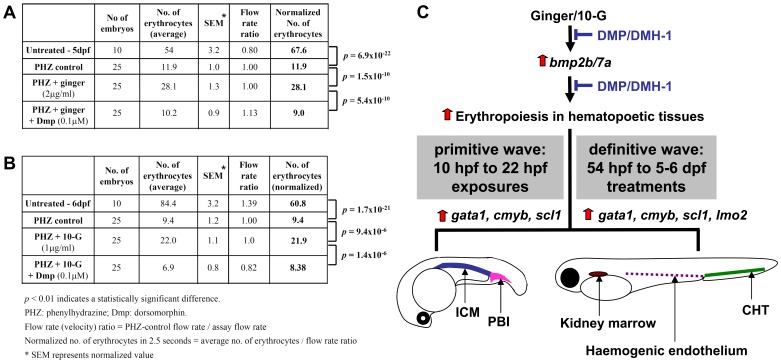
Ginger or 10-G exposure promotes erythrocyte recovery from anemia via a Bmp/Smad signal-dependent mechanism. Bmp/Smad inhibition abolishes the hematopoiesis promoting effect of ginger and 10-G. (A–B) The effect of ginger on hematopoiesis was quantitated in zebrafish embryos after phenylhydrazine (PHZ) induced acute hemolytic anemia, followed by extensive washes and treatments with ginger extract (A) or 10-G (B) with or without dorsomorphin (DMP; 0.1 µM). Ginger and 10-G promote hematopoietic recovery in PHZ treated embryos. Videos of circulating erythrocytes were analyzed and erythrocyte numbers for “PHZ+ginger” and “PHZ+ginger+DMP” assays were calculated and normalized with blood flow (velocity) using the PHZ control value as a reference. Tables summarize the results of one representative experiment. Experiments were repeated 3 times. n = number of embryos analyzed per group. *p* values were determined by using the Student’s t-test. (C) Regions of erythropoiesis promoted by ginger and 10-G are indicated on cartoons of zebrafish embryos at 22 hpf (primitive wave; before circulation), and at 5–6 dpf during the definitive wave of hematopoiesis. DMP-mediated inhibition of Bmp/Smad signal refers to Figures S5, S6, 6 and S7 data. DMH1-mediated inhibition of Bmp/Smad signaling refers to Figures S5, S6 and S8 data. During the primitive wave of hematopoiesis, expression of *gata1* and *Tg(gata1:dsRed)* were increased in the ICM and PBI, as shown in Figure 1, and the hematopoietic progenitor markers *cmyb* and *scl* were up-regulated in the same hematopoietic tissues, as shown in Figure 2. During the definitive wave, *Tg(gata1:dsRed)* circulating cells were promoted at 5/6 dpf upon ginger/10-G exposure (Figures 5 and 6), and the hematopoietic progenitor markers *cmyb*, *scl* and *lmo2* were up-regulated in the CHT/hemogenic endothelium at 6 dpf (*cmyb,* Figure 7) or in the CHT only at 5 dpf (*scl* and *lmo2,* Figure S9).

Since *bmp* expression is up-regulated in response to ginger or 10-G exposure in zebrafish embryos, we asked whether Bmp signaling was essential for ginger-induced hematopoietic recovery by inhibiting its downstream target Bmp-activated Bmp type I receptor kinase. Inhibition of this receptor kinase, using the specific pharmacological antagonist dorsomorphin [40], will block Bmp-mediated Smad phosphorylation. The inhibition of Bmp signaling during gastrulation is not feasible since Bmp signals are crucial to early development [38]; therefore, we treated embryos with dorsomorphin in combination with ginger or 10-G at 54 hpf beyond the time when Bmp activity is essential for dorsal-ventral patterning. The effect of ginger or 10-G on hematopoiesis is completely abolished by a low concentration (0.1 µM) of dorsomorphin in phenylhydrazine-induced anemic zebrafish (Figure 6A and B; Videos S9 and S10). The treatment of control embryos and anemic embryos with dorsomorphin alone has no significant effect on the number of circulating *Tg(gata1:dsRed)* cells (Figure S7). We repeated these analyses using the dorsomorphin analogue DMH-1 (0.1 µM), which exclusively targets the Bmp but not Vegf signaling [42]. We observed the same suppression of ginger-induced erythroid recovery at 5 dpf (Figure S8). Altogether, these quantitative data show that ginger extract or 10-G can boost the hematopoietic recovery from anemia via a Bmp/Smad signaling-dependent mechanism.

**Figure 7:**
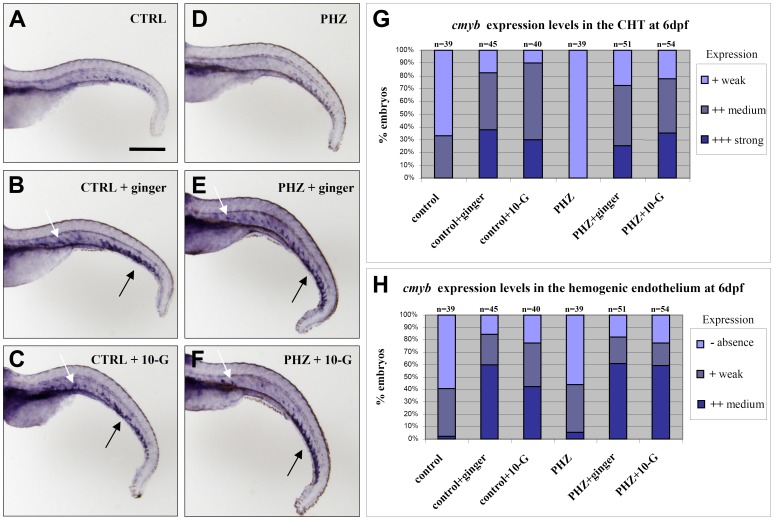
Effect of ginger and 10-G treatments on *c-myb* expression in zebrafish embryos at 6 dpf. (A–C) Ginger (B) or 10-G (C) treatment from 2 dpf to 6 dpf promotes *cmyb* expression in the CHT (black arrows) and the hemogenic endothelium (white arrows) along the ventral wall of the dorsal aorta (AGM equivalent) in the trunk and tail regions of normal embryos. (D–F) In phenylhydrazine-induced anemic embryos, ginger (E) or 10-G (F) treatment similarly promotes *cmyb* expression both in the CHT (black arrows) and the hemogenic endothelium (white arrows). (G–H) Graphical representation of the percentage of embryos showing *cmyb* expression in the CHT (G) and in the hemogenic endothelium (H). CTRL: control; PHZ: phenylhydrazine; n = number of embryos. Scale bar = 500 µm.

As a result, we investigated the hematopoietic progenitors during the second or definitive wave of hematopoiesis at 6 dpf by whole-mount in situ hybridization using the hematopoietic progenitor markers *cmyb, scl* and *lmo2*. When the zebrafish embryos were treated with ginger or 10-G during the period from 54 hpf to 6 dpf, they exhibited stronger expression of *cmyb* along the CHT and the hemogenic endothelium (Figure 7A–C; cartoon Figure 6C) in comparison with control embryos. The same increase in *cmyb* expression in the CHT and the hemogenic endothelium was observed in anemic embryos treated with ginger or 10-G (Figure 7D–F). In addition, ginger exposure also up-regulates the expression of *scl/tal1* and *lmo2* transcription factors in the CHT, especially in anemic zebrafish embryos (Figure S9). *scl* is expressed in progenitors during development and serves as an early marker of hemangioblasts that are already fated to become hematopoietic cells. Lmo2 (LIM domain only 2, also known as Rhombotin-like 1) is required for hematopoietic stem cell differentiation, similar to Scl, which interacts with Lmo2 in a multiple transcription factor complex required for the specification of early blood cells and the regulation of the early hematopoietic program in the developing embryo. Hence, our data suggests that ginger or 10-G treatments not only increase the primitive wave of hematopoiesis (Figures 1 and 2), but also enhance the number of hematopoietic progenitors during the definitive wave (Figures 7 and S9).

**Figure 8:**
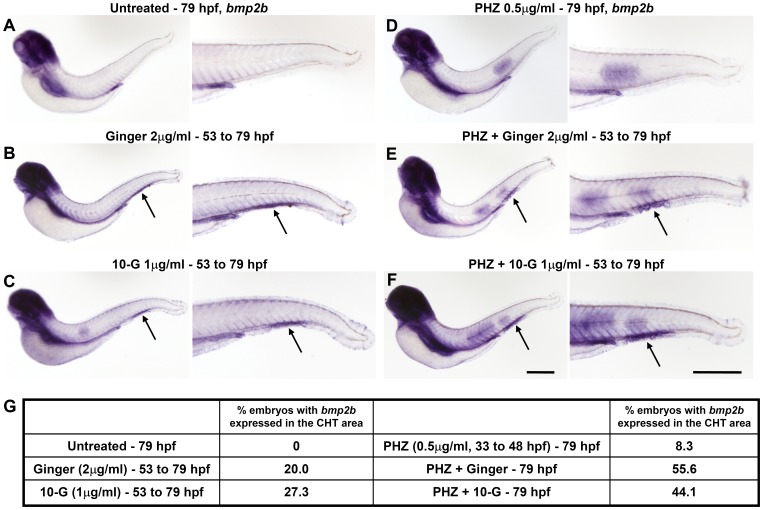
Over-expression of *bmp2b* specifically localized in the CHT area at 79 hpf upon ginger or 10-G exposure in normal and in anemic zebrafish embryos. Whole-mount in situ hybridization of *bmp2b.* (A–C, left) Normal non-anemic control embryos or embryos treated with ginger/10-G. (D–F, right) Anemic control embryos or anemic embryos treated with ginger/10-G. Anemic groups were treated with 0.5 µM PHZ from 33 to 48 hpf. Embryos express *bmp2b* in the CHT region (arrows) following exposure to ginger (B, E) or 10-G (C, F). (G) A table shows the percentage of embryos with *bmp2b* expression in the CHT area at 79 hpf. Scale bars = 420 µm.

**Figure 9:**
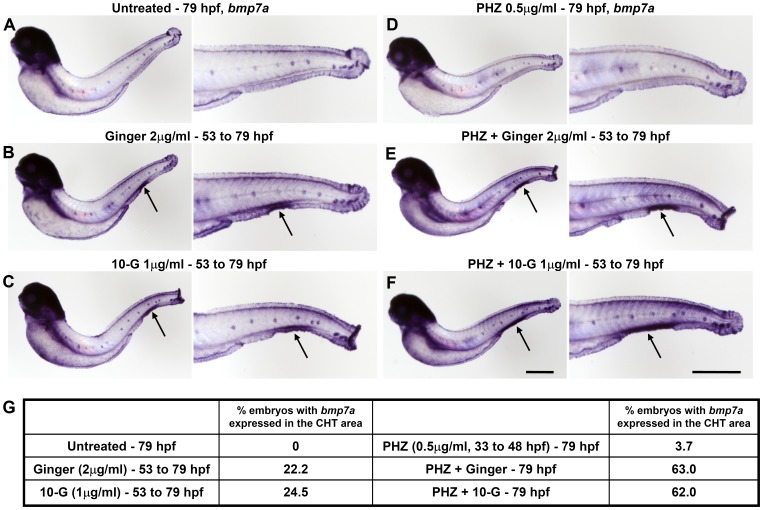
Over-expression of *bmp7a* specifically localized in the CHT region at 79 hpf upon ginger or 10-G exposure in normal and in anemic zebrafish embryos. Whole-mount in situ hybridization of *bmp7a*. (A–C, left) Normal non-anemic control embryos or embryos treated with ginger/10-G. (D–F, right) Anemic control embryos or anemic embryos treated with ginger/10-G. Anemic group were treated with 0.5 µM PHZ from 33 to 48 hpf. Embryos express *bmp7a* in the CHT area (arrows) following exposure to ginger (B, E) or 10-G (C, F). (G) A table shows the percentage of embryos with *bmp7a* expression in the CHT region at 79 hpf. Scale bars = 420 µm.

Altogether, our data are consistent with previous observations in mammals showing that Bmp is necessary for the proliferation of HSCs [1]. The exposure of normal and phenylhydrazine-treated embryos to ginger extract or its active phenolic component, 10-G, at 54 hpf induced the expression of *bmp2b* and *bmp7a* restricted to the CHT region at 3 dpf (Figures 8 and 9 respectively), whereas *bmp* expression is already down-regulated in control embryos at this stage. This ginger-induced up-regulation of *bmp2b/7a* localized in the CHT area is likely to lead to the activation of the Bmp/Smad signaling and the over-expression of hematopoietic progenitor markers such as *cmyb* (Figure 7), *scl/tal1 and lmo2* (Figure S9). Eventually, it increases the number of circulating *Tg(gata1:dsRed)* erythroid cells, which is normally regulated by the Bmp pathway, especially during recovery from chemically-induced anemia (Figures 6 and S8).

## Discussion

Overall, our results demonstrate that ginger extract and its purified component 10-G potentially stimulate both the primitive and definitive waves of hematopoiesis in zebrafish embryos. We also show that the treatment with ginger or 10-G promotes the hematopoietic recovery from phenylhydrazine-induced anemia in this model. Finally, we provide mechanistic evidence that the hematopoiesis-promoting effects of ginger and 10-G are mediated through the modulation of *bmp* expression and Bmp signaling pathway in zebrafish (Figures 4, 8, 9, S5, S6, 6, S7 and S8).

In the literature, *bmp2b* expression patterns have not been documented after 38 hpf by in situ hybridization [43] and *bmp7a* expression data are available until 24 hpf only [44]. In the present study, we show expression of *bmp2b/7a* at 8 hpf and 48 hpf in control embryos and in embryos exposed to ginger/10-G, where we observed an up-regulation of their transcription in the CHT region at 48 hpf (Figure 4). We also illustrated expression of *bmp2b/7a* at 79 hpf in normal and anemic embryos and showed that upon ginger/10-G exposure from 54 hpf, the same ectopic induction of their expression in the area of the CHT can be observed (Figures 8 and 9). Indeed, Wiley et al. have shown that *bmp2b* is expressed in the caudal vein plexus at 32 hpf, but not at 26 hpf or 38 hpf [43], in accordance with our observations in control embryos at 32 hpf (not shown) and 48 hpf (Figures 4, S5, S6).

In zebrafish and other vertebrates, Bmp signal plays an essential role during gastrulation and regulates dorsal-ventral patterning in concert with Nodals, Wnts and Fgfs: high ratio of BMP signaling promotes ventral fates including blood, vasculature and pro-nephritic ducts. In adult fish, kidney marrow is the site of hematopoiesis, Smad-mediated Bmp signaling was also detected in the proximal and distal kidney in *bmp* response element transgenic zebrafish line [45]. The same study also illustrated the normal expression of phosphorylated Smad1/5/8 in 3 dpf larvae at the tip of the tail, as well as ventrally anterior to the proctodeum, thus including the transient hematopoietic tissues during definitive hematopoiesis.

In Mammals, it is now clear that the BMP signaling pathway plays a critical role in the maintenance of HSC potential in the Aorta-Gonad-Mesonephros (AGM) region [11]. Another study confirmed that BMP increases the growth and survival of AGM HSCs in long-term culture [15]. Yet one group showed that BMP7, but not BMP2 or BMP4, improves maintenance of human primitive peripheral blood-derived hematopoietic progenitor cells [2]. Our findings that ginger extract induces *bmp* expression in the area of the transient caudal hematopoietic tissue, *cmyb* in hematopoietic progenitors and *gata1* in erythrocytes and erythroid progenitors of zebrafish embryos are consistent with another study, as Bmp is well known for its bone promoting ability, where mouse fetuses exposed to ginger extract were heavier and had more advanced skeletal development than control embryos [46]. Therefore, future studies to explore the outcomes of various combinations of ginger components on hematopoiesis in mammalian models may provide new insights for nutraceutical development to promote erythropoiesis for treating pathological anemia.

## Materials and Methods

### Zebrafish Husbandry

Zebrafish AB transgenic strains *Tg(gata1:dsRed)* [47] and *Tg(flk1:GFP)* [48] have been described previously; embryos were staged and maintained according to NCCU IACUC guidelines. Zebrafish embryos with or without ginger or gingerol components were incubated at 28.5°C in 0.3X Danieau’s solution (19.3 mM NaCl, 0.23 mM KCl, 0.13 mM MgSO_4_, 0.2 mM Ca(NO_3_)_2_, 1.7 mM HEPES, pH 7.0) containing 30 µg/ml phenylthiourea (PTU, added after late gastrulation stages) to inhibit pigmentation. Following or prior to exposure to ginger or phenolic compounds, embryos were washed, dechorionated and anaesthetized before observations, picture acquisitions, or fixation in 4% paraformaldehyde (PFA).

### Whole-mount in situ Hybridization

The in situ hybridization procedure has been described previously [49] as probes: *cmyb* [18], *gata1* [33], *gata2* [35], *eve1* [35], *scl* [21], *lmo2* [22], *bmp2b* [50], *bmp4* [51], *bmp7a* [51].

### Fluorescent Microscopy

Imaging was performed using an Olympus MVX10 MacroView Fluorescence Microscope (Olympus, Center Valley, PA) with Hamamatsu C9300-221 high-speed digital CCD camera (Hamamatsu City, Japan). For time-lapse imaging, transgenic *Tg(gata1:dsRed)* fluorescent embryos were anaesthetized in tricaine and imaged at 5 or 6 dpf. Picture acquisition parameters were kept constant to allow direct comparisons. Raw data were analyzed using MetaMorph TL for Olympus software (Olympus, Center Valley, PA) and exported in QuickTime format.

### Quantitation of Erythrocytes

The effect of ginger and 10-G on hematopoiesis was quantified after induction of acute hemolytic anemia using 0.5 µg/ml phenylhydrazine (PHZ). PHZ was added into the incubation medium (0.3X Danieau’s solution) of 33 hpf *Tg(gata1:dsRed)* positive embryos, followed by extensive washes at 48 hpf. Embryos were then incubated with ginger or 10-G and/or dorsomorphin (DMP; 0.1 µM). Videos of circulating erythrocytes within the caudal artery were taken at 5 or 6 dpf under a fluorescence microscope. The videos of circulating erythrocytes were analyzed in a minimum of 25 embryos per experimental condition by counting the number of *Tg(gata1:dsRed)* fluorescent cells entering/exiting the artery section (100 frames in 2.5 seconds; 327 µm distance). Average calculated numbers of erythrocytes were normalized with blood flow (velocity) ratio. In order to perform this normalization, three independent cells were chosen arbitrarily to count the number of frames required for each cell to cross the filmed portion of the dorsal aorta (327 µm x 246 µm). The average number of frames was then used to calculate the flow rate and to normalize the number of *Tg(gata1:dsRed)* cells in the assay condition with the PHZ control using the flow rate ratio (PHZ control flow rate/assay flow rate). This analysis was performed for 25–30 embryos per experimental condition, the average was calculated for all parameters and Student’s t-tests were used to determine statistical significances. Data presented for fluorescent erythrocyte counts in *Tg(gata1:dsRed)* transgenic embryos are mean ± SEM. To analyze the difference between two groups, *p* values were determined by using the Student’s t-test. *p*<0.01 was considered statistically significant. The quantitation procedure was repeated in 3 independent experiments for ginger and for 10-G using DMP to inhibit Bmp/Smad signaling. It was also repeated in 2 independent experiments using the DMP analogue DMH-1 (0.1 µM).

### Isolation of the Major Gingerols and Shogaols from Ginger Extract

6-, 8-, and 10-gingerol and 6-, 8-, and 10-shogaol were purified from ginger extract in our laboratory using previously reported methods with slight modifications [31]. In brief, the ginger extract was chromatographed on a Diaion HP-20 column eluted first with 50% aqueous ethanol to obtain fraction A, followed by 75% aqueous ethanol to obtain fractions B and C, and finally 95% aqueous ethanol to obtain fraction D. Following our previous methods, 6-gingerol was purified from fraction A, 6-shogaol and 8- and 10-gingerol were purified from fraction B, and 8- and 10-shogaol were purified from fraction C. The purification procedure was guided by thin layer chromatography (TLC) and high-performance liquid chromatography (HPLC) analysis. The structures of these six compounds were confirmed by ^1^H and ^13^C NMR analysis [31].

### Incubation with Ginger and its Components

Ginger extract, 6-, 8-, 10-gingerol and 6-, 8-, 10-shogaols were dissolved in dimethyl sulfoxide (DMSO) to prepare the stock solutions. Ginger extract and its purified compounds were diluted in 0.3X Danieau’s solution containing PTU. The final concentration of DMSO in experiments was less than or equal to 0.05% (v/v; 0.002 to 0.05%), which has no effect on differentiation nor proliferation of BB88/TIB-55 cells or zebrafish embryonic development.

### Cell Culture

The murine erythroleukemia (BB88/TIB-55) cell line [52]–[53] (ATCC, Rockville, MD) was cultured in ATCC-formulated RPMI-1640 supplemented with 50 µM 2-mercaptoethanol, 10% ATCC FBS and antibiotics. Cells were cultured at 37°C in a humidified atmosphere of air with 5% CO_2_. BB88 cells (erythroblasts) were induced to differentiate into erythrocytes by transiently adding 1.8% dimethyl sulfoxide (DMSO; positive control) or ginger extract (5, 10 and 20 µg/ml; assays). 0.0125%, 0.025% and 0.05% DMSO were used as additional negative control for ginger extract.

### Benzidine Staining of Hemoglobins

1 ml of benzidine stock solution (2 g/L benzidine dihydrochloride in double-distilled water containing 2.9% (v/v) glacial acetic acid) was mixed with 20 µl of 33% H_2_O_2_ to prepare the working solution. Cells were mixed with the benzidine working solution at a 1∶1 (v/v) ratio, incubated 2–3 minutes, and hemoglobin positive cells were scored using a hemocytometer (blue cells). Trypan blue exclusion viability analyses were performed in parallel.

## Supporting Information

Figure S1 **HPLC profile of ginger extract and structures of the major gingerols and shogaols.** Left panel: HPLC. Right panel: chemical structures.
(TIF)

Figure S2 **Ginger induces erythrocyte differentiation in mouse erythroblasts.** (A) Graph representing the number of proliferating cells after treatment with ginger, as determined by trypan blue exclusion counts using a hemocytometer (viabilities were 72–83% after 5 days in culture). (B) Graph representing the number of differentiated erythroblasts using benzidine staining of hemoglobins. Cells were exposed continuously to ginger extract (5, 10 and 20 µg/ml) for 5 days. Additional controls for ginger include continuous incubation with 0.0125, 0.025 and 0.05% DMSO, representing the final solvent concentrations in the assays. The experiments were performed in triplicates for SEM determinations. *p* values were determined using the Student′s t-test. 1.8% DMSO (*p* = 3.1×10^−4^), 5 µg/ml ginger (*p* = 1.7×10^−2^) 10 µg/ml ginger (*p* = 7.1×10^−3^), 20 µg/ml ginger (*p* = 2.7×10^−5^). This experiment was repeated 3 times independently with similar results.
(TIF)

Figure S3 ***bmp4*** **expression in late gastrulae exposed to ginger/10-G.** Whole mount in situ analysis of *bmp* expression after treatment with ginger/10-G. The *bmp4* expression pattern at 75% epiboly was not affected by short-term treatment with ginger/10-G from sphere (4 hpf) to 75% epiboly (8 hpf) stages during early development. Embryos are oriented with the dorsal side to the right. Scale bar = 250 µm.
(TIF)

Figure S4 **Ginger treatment of zebrafish embryos does not affect the expression of** ***chd*** **and** ***fgf8*** **.** Whole mount in situ of *chordin* (*chd*) and *fgf8* after treatment with ginger/10-G. Normal expression patterns of both *chd* at the dorsal margin (left panel) and *fgf8* at the dorsal and ventral margins (right panel) at the shield stage after treatment with ginger/10-G. Lateral and animal views of representative embryos, with the dorsal side (D) to the right, ventral (V) to the left. Scale bars = 200 µm.
(TIF)

Figure S5 **Bmp antagonists, that inhibit the canonical BMP-Smad signaling pathway, suppress the ginger-induced** ***bmp2b*** **expression in the region of the developing CHT.** Whole mount in situ hybridization of *bmp2b* expression in zebrafish embryos after treatment with Bmp inhibitors and/or ginger/10-G from 10 to 48 hpf. (A) A control embryo. (B) Zebrafish embryos treated with ginger (5 µg/ml). (C–D) Ginger (5 µg/ml) and Dorsomorphin/DMP, 0.1 and 2 µM. (E–G) Ginger (5 µg/ml) and LDN193189, 0.1, 0.5 and 1 µM. (H) Ginger (5 µg/ml) and DMH1, 0.1 µM. (I) Analyses of *bmp2b* expression localized in the CHT area (table). Scale bars = 300 µm.
(TIF)

Figure S6 **Bmp/Smad signaling antagonists inhibit the ginger-induced** ***bmp7a*** **expression in the area of the developing CHT.** Whole-mount in situ hybridization of *bmp7a* in zebrafish embryos, after treatment with Bmp inhibitors and/or ginger/10-G from 10 to 48 hpf. (A) A control embryo. (B) Zebrafish embryos treated with ginger (5 µg/ml). (C–D) Ginger (5 µg/ml) and dorsomorphin/DMP, 0.1 and 2 µM. (E–G) Ginger (5 µg/ml) and LDN193189, 0.1, 0.5 and 1 µM. (H) Ginger (5 µg/ml) and DMH1, 0.1 µM. (I) Analyses of *bmp7a* expression localized in the CHT region (table). Scale bars = 300 µm.
(TIF)

Figure S7 **Absence of effect for dorsomorphin treatment of normal and anemic zebrafish embryos on the number of circulating erythrocytes.** Table shows the results obtained using the effective concentration of dorsomorphin (0.1 µM). Dorsomorphin alone had no significant effect on the number of circulating erythrocytes within the caudal dorsal aorta, after quantification at 5 dpf, described in Figures 5 and 6.
(TIF)

Figure S8 **Inhibition of Bmp/Smad signal using the dorsomorphin analogue DMH-1 abolishes the stimulating effect of ginger on erythrocyte recovery from PHZ-induced anemia.** Table summarizing the results of treating zebrafish embryos with DMH-1, which specifically targets the Bmp/Smad signal transduction without affecting the Vegf signaling. Experiments were repeated 2 times. n = number of embryos analyzed per group. Procedure as described in Figure 6A; embryos with extremely low blood flow were excluded from analysis. *p* values were determined by using the Student’s t-test. Erythrocyte numbers were generally higher than in Figure 6A, likely due to a faster recovery from PHZ-induced anemia.
(TIF)

Figure S9 **Up-regulation of hematopoietic progenitor markers** ***scl/tal1*** **and** ***lmo2*** **in the CHT at 5** **dpf following ginger treatment in anemic zebrafish embryos.** (A–B) Whole-mount in situ hybridization of *scl/tal1* (A) and *lmo2* (B) showing over-expression of these hematopoietic progenitor markers in the CHT (arrows) of anemic embryos treated with ginger extract. Scale bars = 500 µm.
(TIF)

Video S1 **Video of untreated normal zebrafish embryo at 5 dpf showing bright field and** ***Tg(gata1:dsRed)*** **fluorescent erythrocytes.** The box area highlighted the dorsal aorta with blood flow towards the tail. Video length: 3.0 seconds at 40 frames/second.
(MOV)

Video S2 **Video of untreated normal zebrafish embryo at 6 dpf showing circulating** ***Tg(gata1:dsRed)*** **fluorescent erythrocytes within the caudal artery.** Video length: 2.5 seconds at 40 frames/second.
(MOV)

Video S3 **Video of phenylhydrazine-induced anemic zebrafish embryo at 5 dpf showing highly reduced number of circulating** ***Tg(gata1:dsRed)*** **fluorescent erythrocytes within the caudal artery.** Embryos were treated with phenylhydrazine (0.5 µg/ml) from 33 hpf to 48 hpf and then washed extensively. Video length: 2.5 seconds at 40 frames/second.
(MOV)

Video S4 **Video of phenylhydrazine-induced anemic zebrafish embryo treated with ginger at 5 dpf showing circulating** ***Tg(gata1:dsRed)*** **fluorescent erythrocytes within the caudal artery.** Embryos were treated with phenylhydrazine (0.5 µg/ml) from 33 hpf to 48 hpf and then washed extensively. The anemic embryos were subsequently treated with ginger extract (2 µg/ml) from 54 hpf onward (as compared to Video S3). Video length: 2.5 seconds at 40 frames/second.
(MOV)

Video S5 **Video of phenylhydrazine-induced anemic zebrafish embryo at 6 dpf showing circulating** ***Tg(gata1:dsRed)*** **fluorescent erythrocytes within the caudal artery.** Embryos were treated with phenylhydrazine (0.5 µg/ml) from 33 hpf to 48 hpf and then washed extensively. Video length: 2.5 seconds at 40 frames/second.
(MOV)

Video S6 **Video of phenylhydrazine-induced anemic zebrafish embryo treated with 10-G at 6 dpf showing highly reduced number of circulating** ***Tg(gata1:dsRed)*** **fluorescent erythrocytes within the caudal artery.** Embryos were treated with phenylhydrazine (0.5 µg/ml) from 33 hpf to 48 hpf and then washed extensively. The anemic embryos were subsequently treated with 10-G (1 µg/ml) from 54 hpf onward (as compared to Video S5). Video length: 2.5 seconds at 40 frames/second.
(MOV)

Video S7 **Video of normal zebrafish embryo at 3 dpf showing circulating** ***Tg(gata1:dsRed)*** **fluorescent erythrocytes within the caudal artery.** Video length: 2.5 seconds at 40 frames/second.
(MOV)

Video S8 **Video of phenylhydrazine-induced anemia of zebrafish embryo at 3 dpf showing no circulating** ***Tg(gata1:dsRed)*** **fluorescent erythrocytes within the caudal artery.** Embryos were treated with phenylhydrazine (0.5 µg/ml) from 33 hpf to 48 hpf and then washed extensively (as compared to Video S7). Video length: 2.5 seconds at 40 frames/second.
(MOV)

Video S9 **Video of phenylhydrazine-induced anemic zebrafish embryo treated with ginger + DMP at 5 dpf showing circulating** ***Tg(gata1:dsRed)*** **fluorescent erythrocytes within the caudal artery.** Embryos were treated with phenylhydrazine (0.5 µg/ml) from 33 hpf to 48 hpf and then washed extensively. The anemic embryos were subsequently treated with ginger extract (2 µg/ml) and dorsomorphin (DMP; 0.1 µM) from 54 hpf onward (as compared to Video S4). Video length: 2.5 seconds at 40 frames/second.
(MOV)

Video S10 **Video of phenylhydrazine-induced anemic zebrafish embryo treated with 10-G + DMP at 6 dpf showing circulating** ***Tg(gata1:dsRed)*** **fluorescent erythrocytes within the caudal artery.** Embryos were treated with phenylhydrazine (0.5 µg/ml) from 33 hpf to 48 hpf and then washed extensively. The anemic embryos were subsequently treated with 10-G (1 µg/ml) and dorsomorphin (DMP; 0.1 µM) from 54 hpf onward (as compared to Video S6). Video length: 2.5 seconds at 40 frames/second.
(MOV)
