# Supplementary figures and images for: Ginger Stimulates Hematopoiesis via Bmp Pathway in Zebrafish
Source: PLoS One. 2012 Jun 25;7(6):e39327. doi: 10.1371/journal.pone.0039327 (PMC3382625; doi:10.1371/journal.pone.0039327)

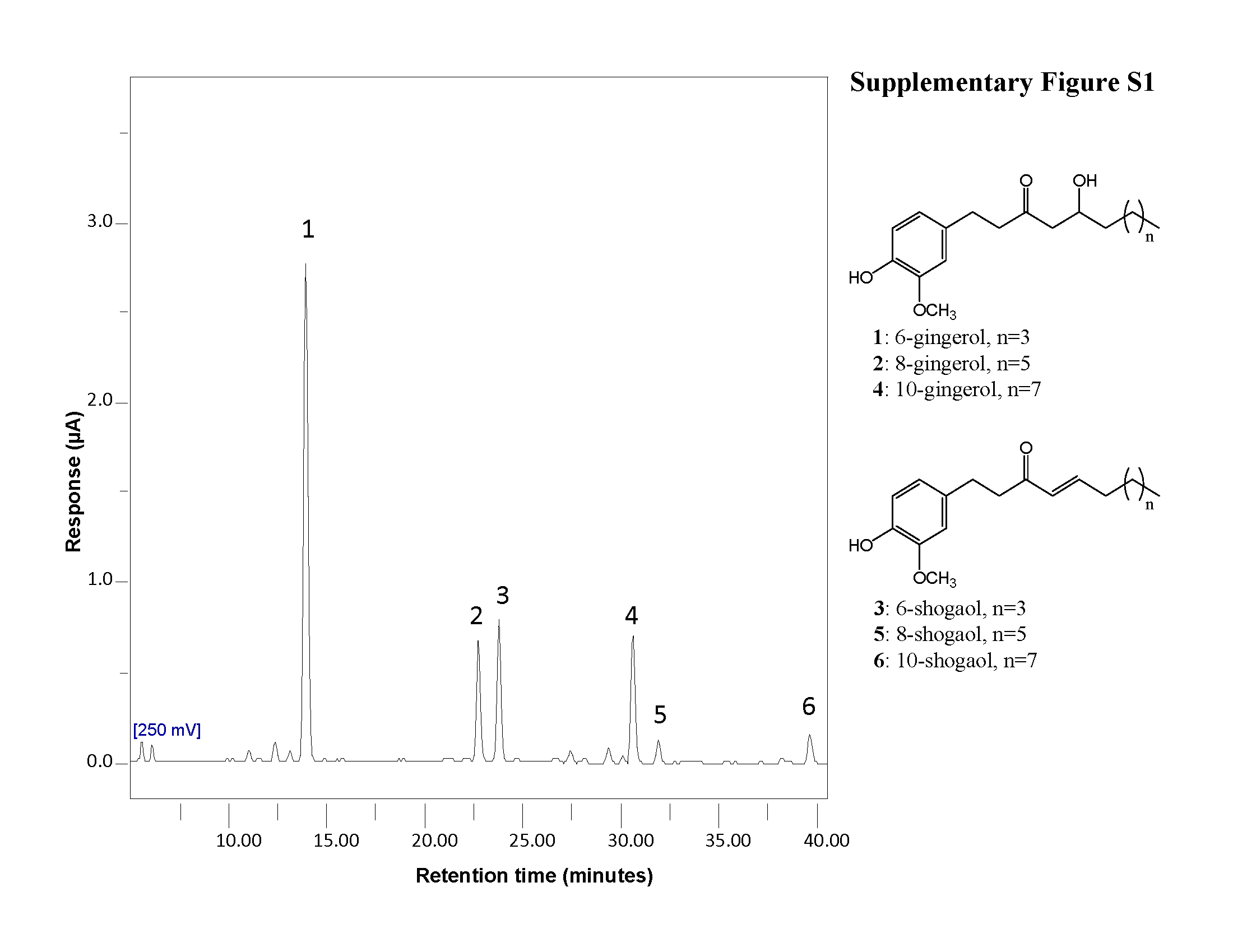

Supplement: Figure S1 — HPLC profile of ginger extract and structures of the major gingerols and shogaols. Left panel: HPLC. Right panel: chemical structures. (TIF) [file pone.0039327.s001.tif]

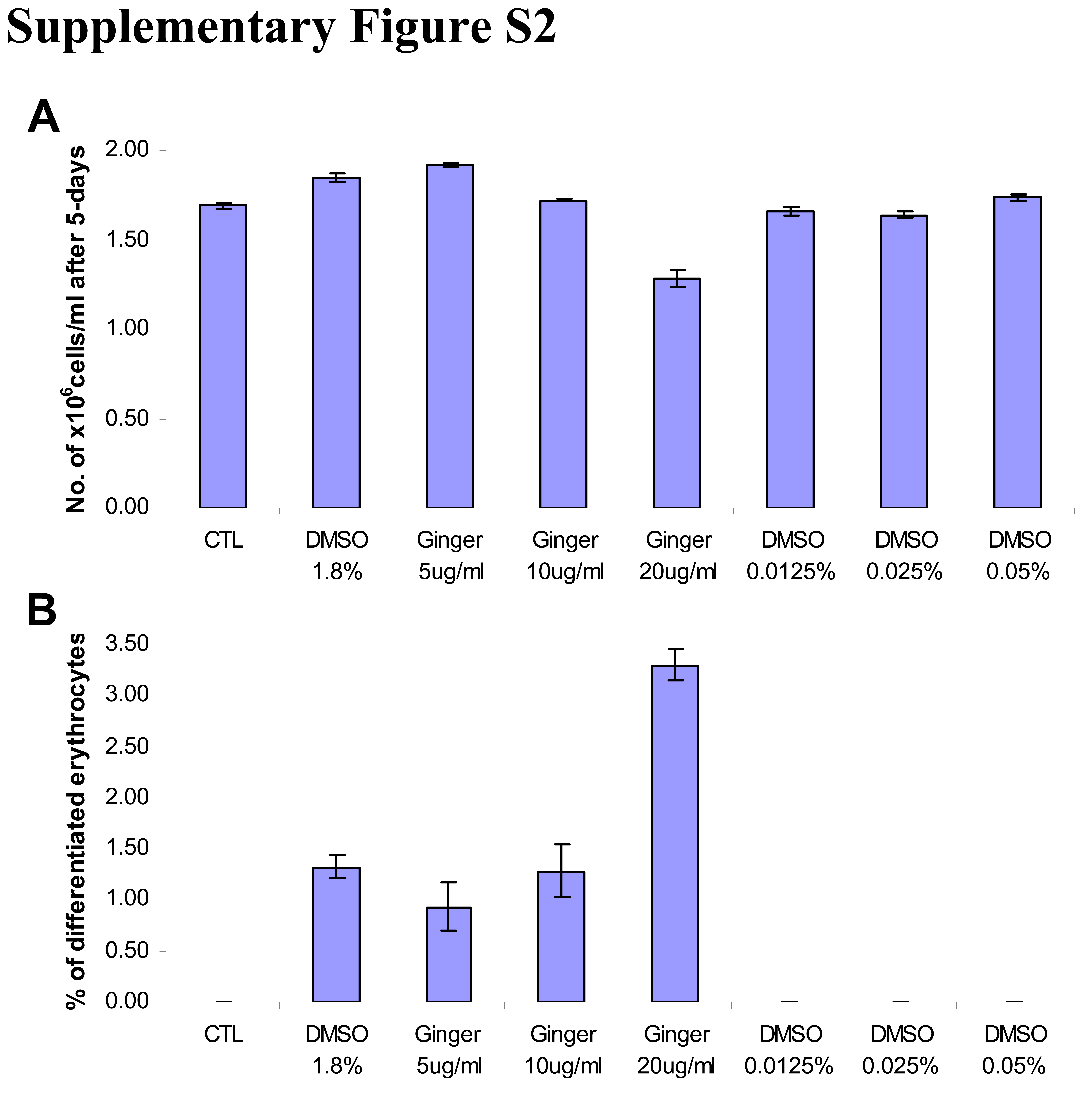

Supplement: Figure S2 — Ginger induces erythrocyte differentiation in mouse erythroblasts. (A) Graph representing the number of proliferating cells after treatment with ginger, as determined by trypan blue exclusion counts using a hemocytometer (viabilities were 72–83% after 5 days in culture). (B) Graph representing the number of differentiated erythroblasts using benzidine staining of hemoglobins. Cells were exposed continuously to ginger extract (5, 10 and 20 µg/ml) for 5 days. Additional controls for ginger include continuous incubation with 0.0125, 0.025 and 0.05% DMSO, representing the final solvent concentrations in the assays. The experiments were performed in triplicates for SEM determinations. p values were determined using the Student′s t-test. 1.8% DMSO (p = 3.1×10^−4^), 5 µg/ml ginger (p = 1.7×10^−2^) 10 µg/ml ginger (p = 7.1×10^−3^), 20 µg/ml ginger (p = 2.7×10^−5^). This experiment was repeated 3 times independently with similar results. (TIF) [file pone.0039327.s002.tif]

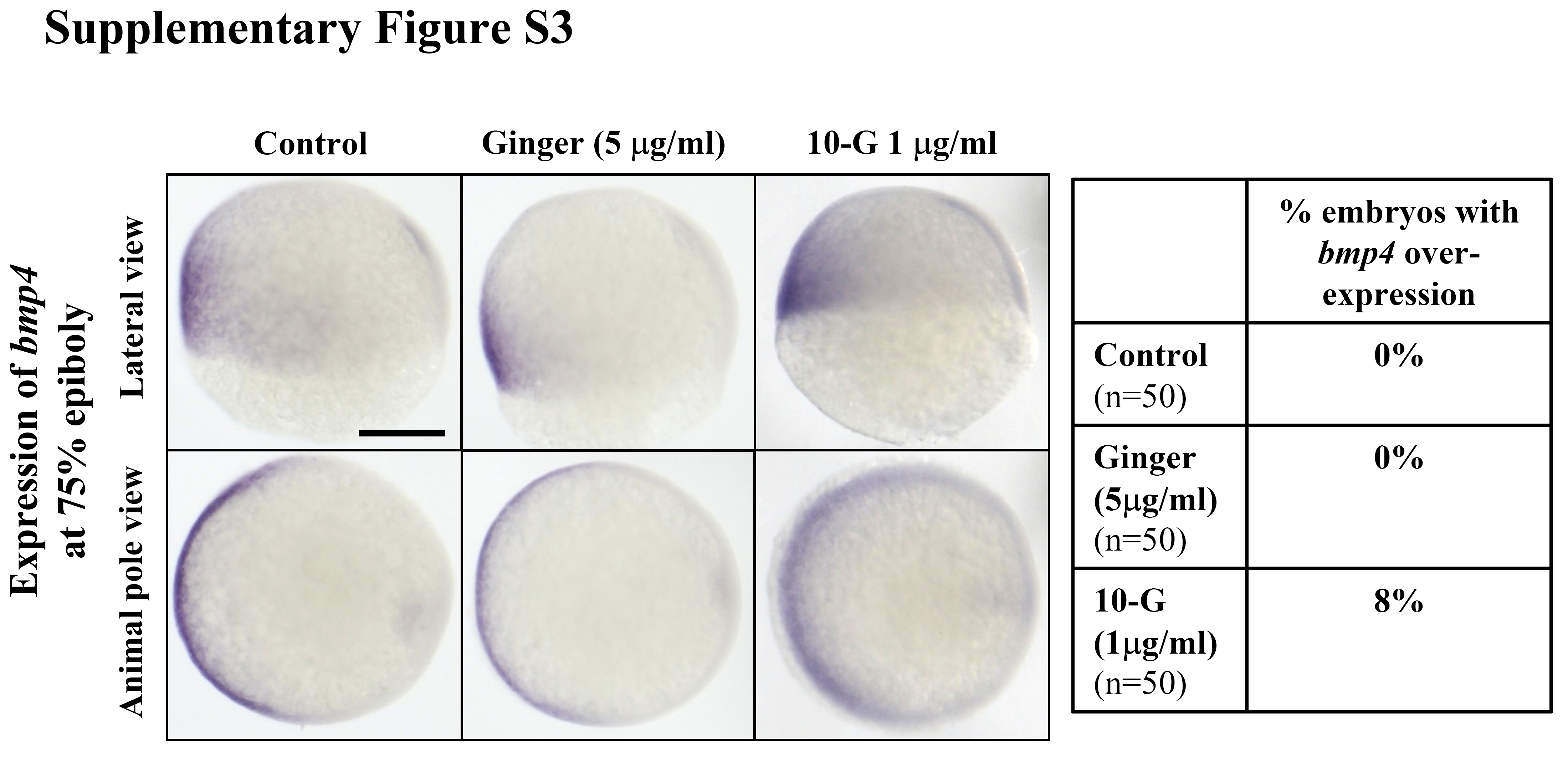

Supplement: Figure S3 — bmp4 expression in late gastrulae exposed to ginger/10-G. Whole mount in situ analysis of bmp expression after treatment with ginger/10-G. The bmp4 expression pattern at 75% epiboly was not affected by short-term treatment with ginger/10-G from sphere (4 hpf) to 75% epiboly (8 hpf) stages during early development. Embryos are oriented with the dorsal side to the right. Scale bar = 250 µm. (TIF) [file pone.0039327.s003.tif]

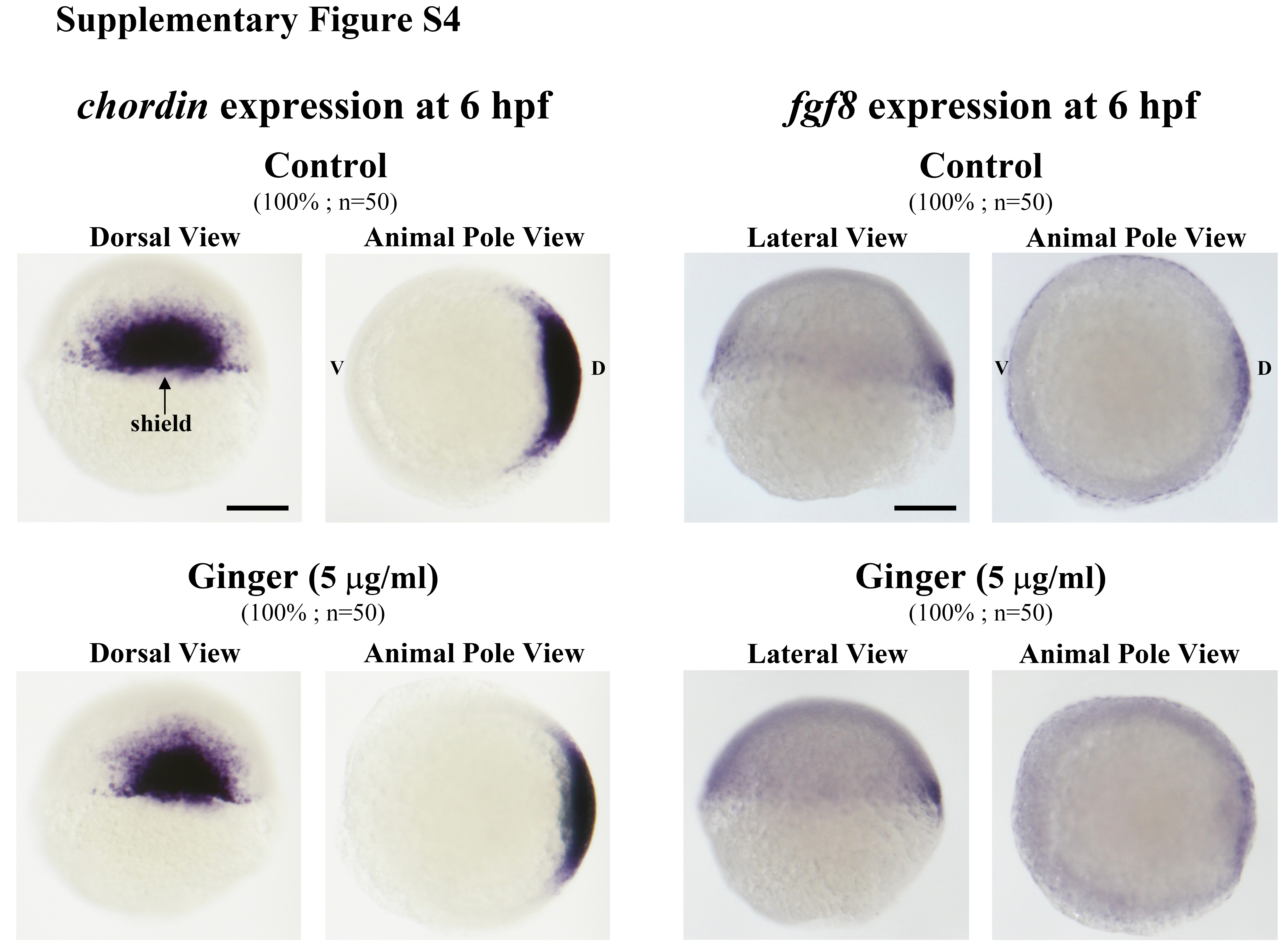

Supplement: Figure S4 — Ginger treatment of zebrafish embryos does not affect the expression of chd and fgf8 . Whole mount in situ of chordin (chd) and fgf8 after treatment with ginger/10-G. Normal expression patterns of both chd at the dorsal margin (left panel) and fgf8 at the dorsal and ventral margins (right panel) at the shield stage after treatment with ginger/10-G. Lateral and animal views of representative embryos, with the dorsal side (D) to the right, ventral (V) to the left. Scale bars = 200 µm. (TIF) [file pone.0039327.s004.tif]

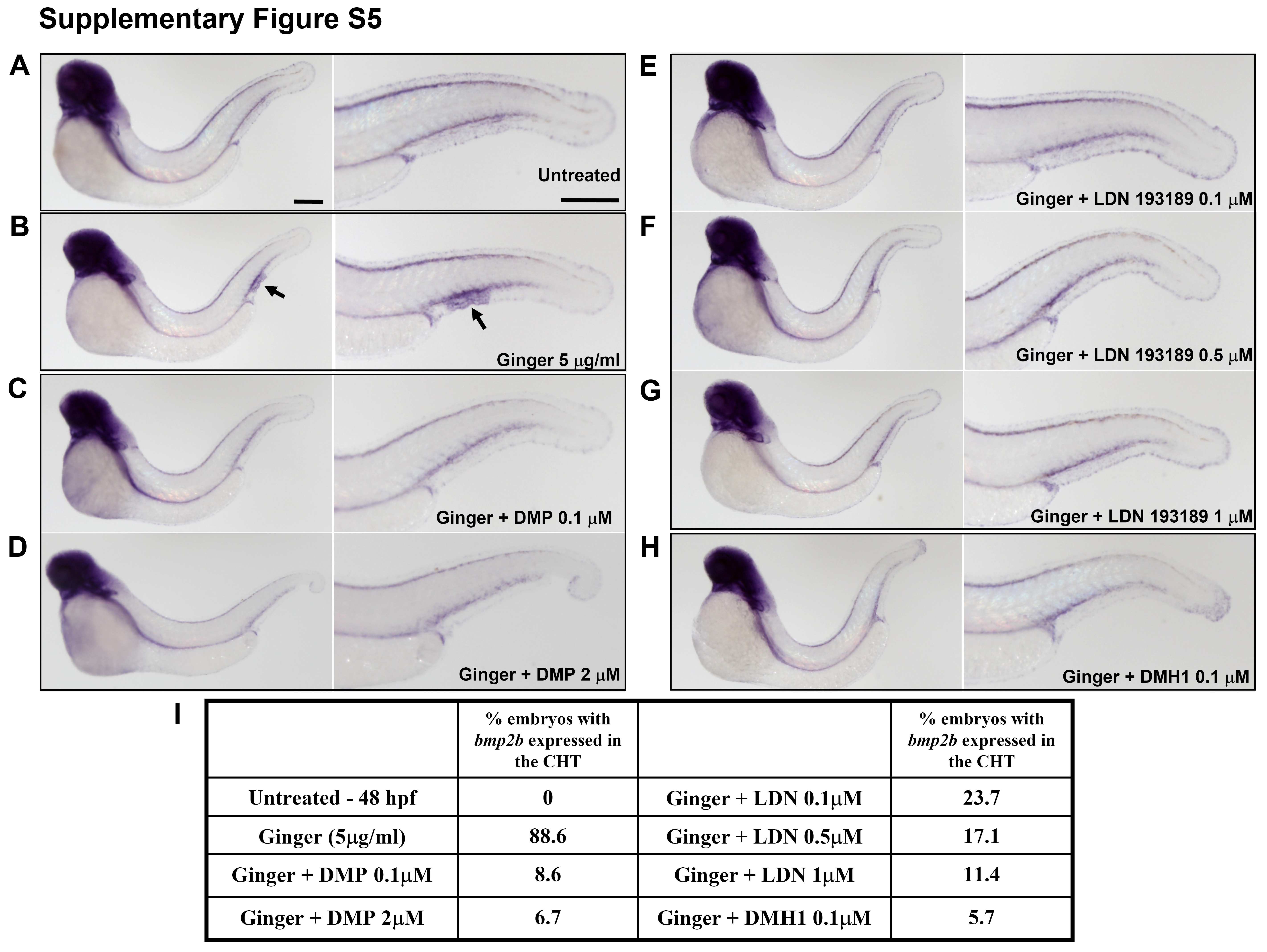

Supplement: Figure S5 — Bmp antagonists, that inhibit the canonical BMP-Smad signaling pathway, suppress the ginger-induced bmp2b expression in the region of the developing CHT. Whole mount in situ hybridization of bmp2b expression in zebrafish embryos after treatment with Bmp inhibitors and/or ginger/10-G from 10 to 48 hpf. (A) A control embryo. (B) Zebrafish embryos treated with ginger (5 µg/ml). (C–D) Ginger (5 µg/ml) and Dorsomorphin/DMP, 0.1 and 2 µM. (E–G) Ginger (5 µg/ml) and LDN193189, 0.1, 0.5 and 1 µM. (H) Ginger (5 µg/ml) and DMH1, 0.1 µM. (I) Analyses of bmp2b expression localized in the CHT area (table). Scale bars = 300 µm. (TIF) [file pone.0039327.s005.tif]

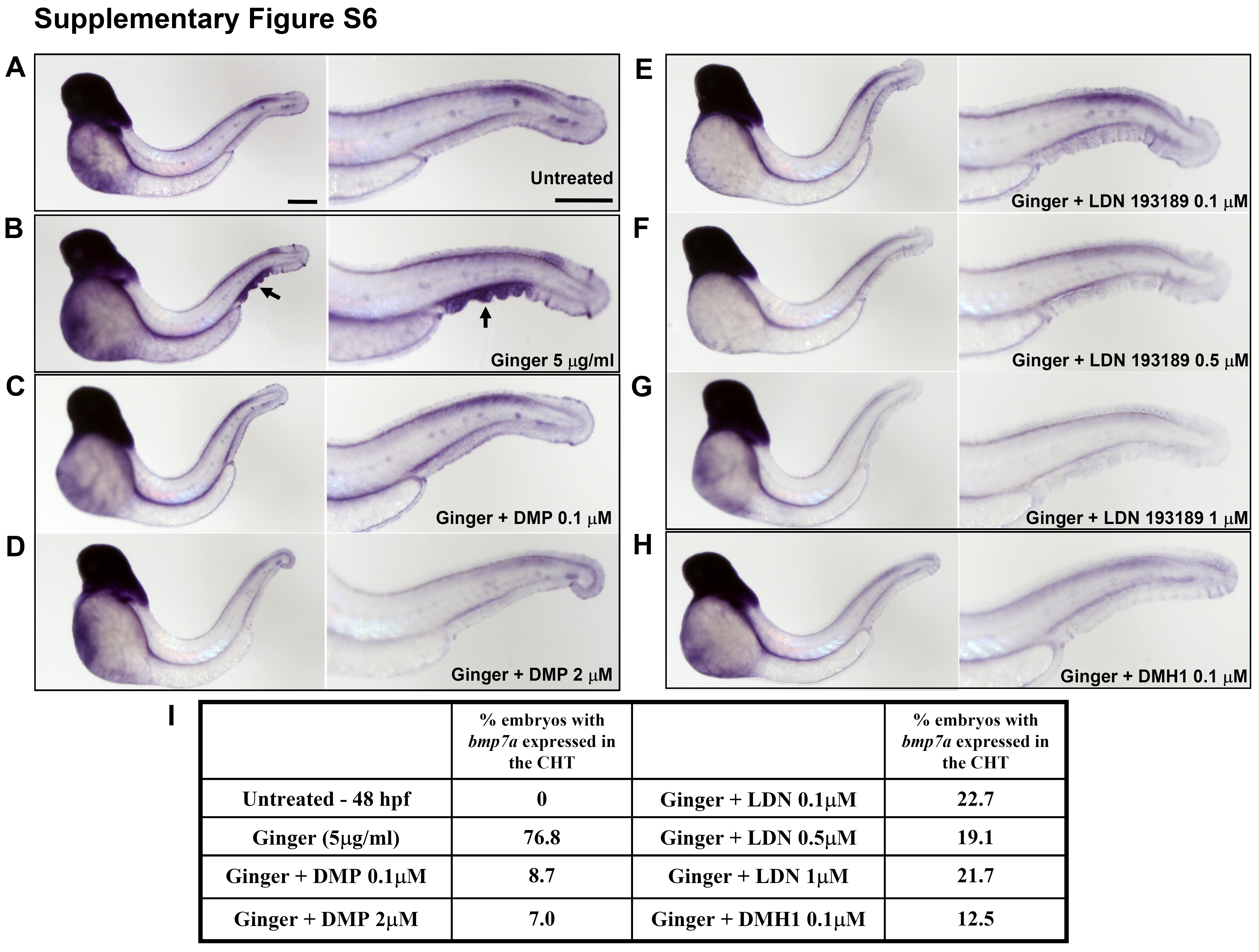

Supplement: Figure S6 — Bmp/Smad signaling antagonists inhibit the ginger-induced bmp7a expression in the area of the developing CHT. Whole-mount in situ hybridization of bmp7a in zebrafish embryos, after treatment with Bmp inhibitors and/or ginger/10-G from 10 to 48 hpf. (A) A control embryo. (B) Zebrafish embryos treated with ginger (5 µg/ml). (C–D) Ginger (5 µg/ml) and dorsomorphin/DMP, 0.1 and 2 µM. (E–G) Ginger (5 µg/ml) and LDN193189, 0.1, 0.5 and 1 µM. (H) Ginger (5 µg/ml) and DMH1, 0.1 µM. (I) Analyses of bmp7a expression localized in the CHT region (table). Scale bars = 300 µm. (TIF) [file pone.0039327.s006.tif]

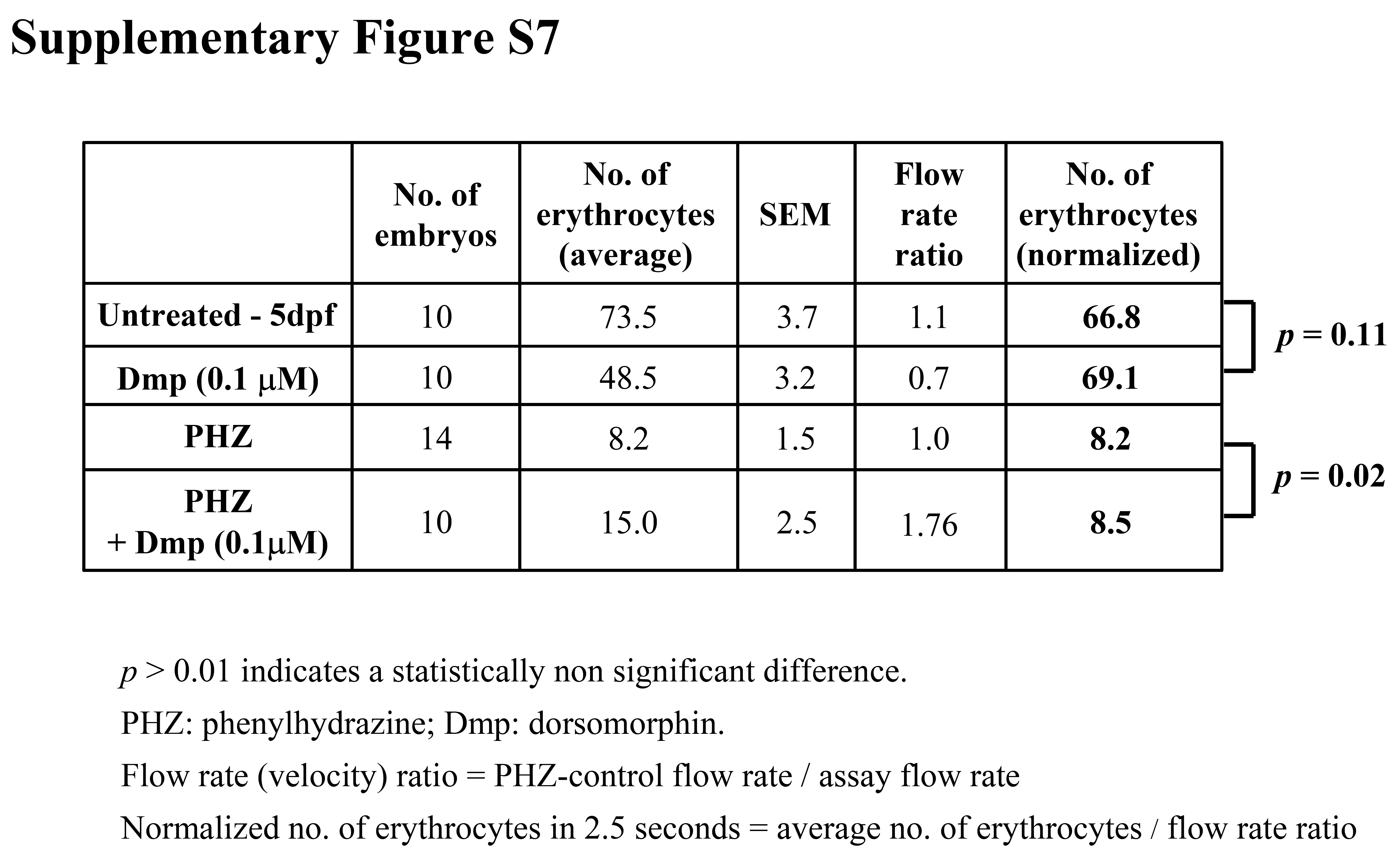

Supplement: Figure S7 — Absence of effect for dorsomorphin treatment of normal and anemic zebrafish embryos on the number of circulating erythrocytes. Table shows the results obtained using the effective concentration of dorsomorphin (0.1 µM). Dorsomorphin alone had no significant effect on the number of circulating erythrocytes within the caudal dorsal aorta, after quantification at 5 dpf, described in Figures 5 and 6. (TIF) [file pone.0039327.s007.tif]

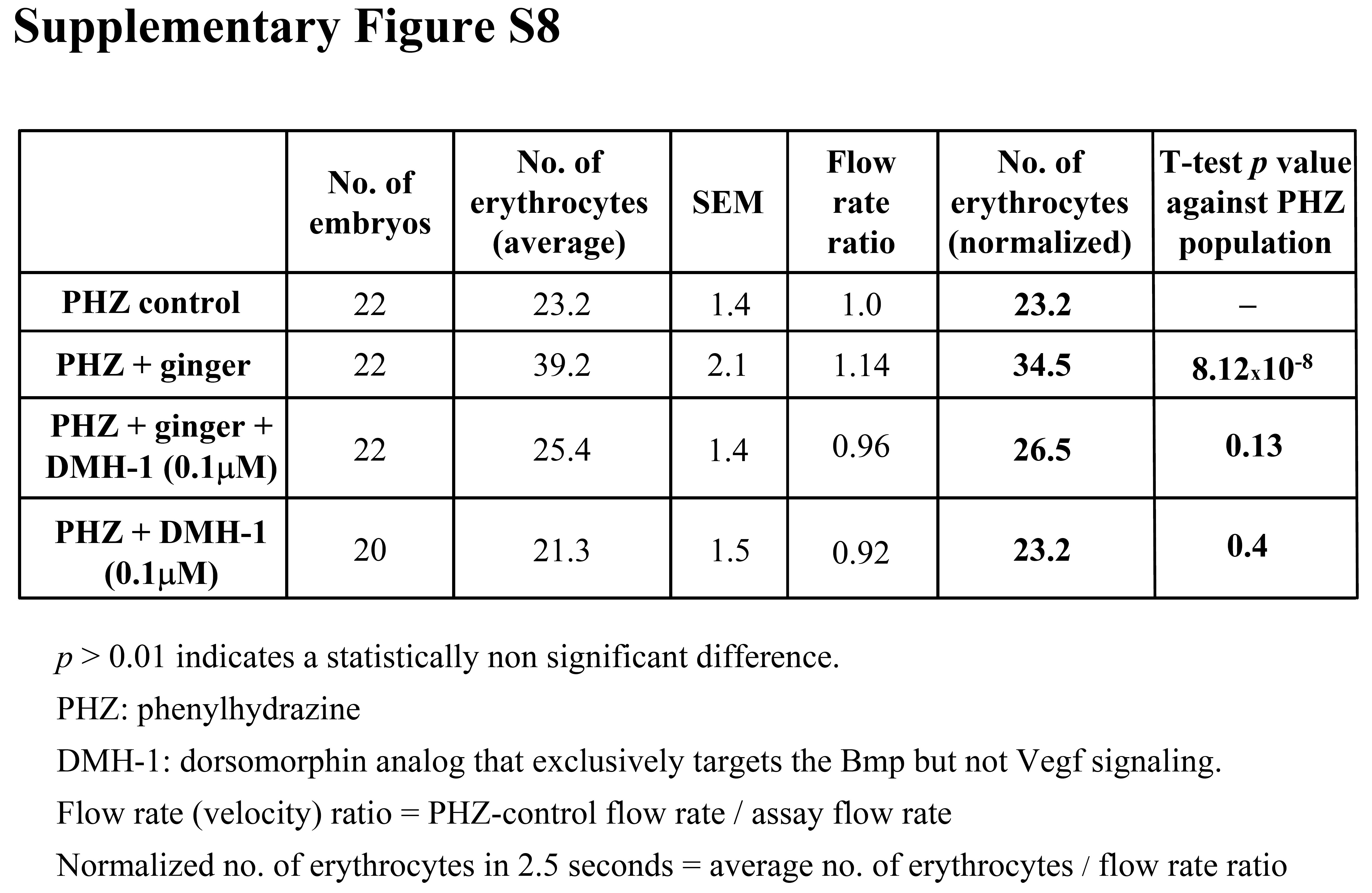

Supplement: Figure S8 — Inhibition of Bmp/Smad signal using the dorsomorphin analogue DMH-1 abolishes the stimulating effect of ginger on erythrocyte recovery from PHZ-induced anemia. Table summarizing the results of treating zebrafish embryos with DMH-1, which specifically targets the Bmp/Smad signal transduction without affecting the Vegf signaling. Experiments were repeated 2 times. n = number of embryos analyzed per group. Procedure as described in Figure 6A; embryos with extremely low blood flow were excluded from analysis. p values were determined by using the Student’s t-test. Erythrocyte numbers were generally higher than in Figure 6A, likely due to a faster recovery from PHZ-induced anemia. (TIF) [file pone.0039327.s008.tif]

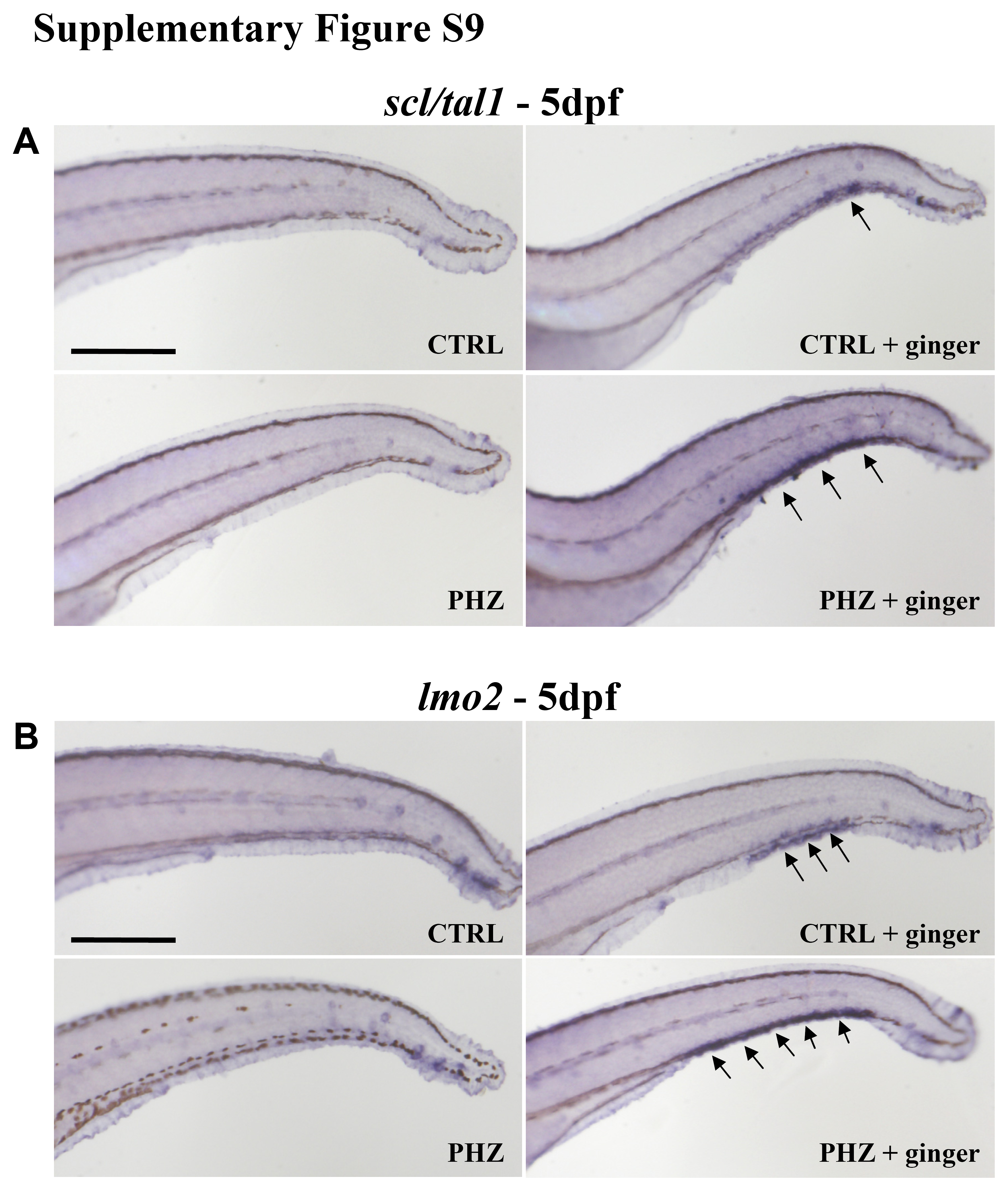

Supplement: Figure S9 — Up-regulation of hematopoietic progenitor markers scl/tal1 and lmo2 in the CHT at 5 dpf following ginger treatment in anemic zebrafish embryos. (A–B) Whole-mount in situ hybridization of scl/tal1 (A) and lmo2 (B) showing over-expression of these hematopoietic progenitor markers in the CHT (arrows) of anemic embryos treated with ginger extract. Scale bars = 500 µm. (TIF) [file pone.0039327.s009.tif]
